# Bacteria: Potential Make-or-Break Determinants of Celiac Disease

**DOI:** 10.3390/ijms25042090

**Published:** 2024-02-08

**Authors:** Ana Roque, Sónia Gonçalves Pereira

**Affiliations:** Center for Innovative Care and Health Technology (ciTechCare), School of Health Sciences, Polytechnic of Leiria, 2410-541 Leiria, Portugal; ana.g.roque@ipleiria.pt

**Keywords:** bacteria, microbiota, host–microbe interactions, disease mechanisms, celiac disease

## Abstract

Celiac disease is an autoimmune disease triggered by dietary gluten in genetically susceptible individuals that primarily affects the small intestinal mucosa. The sole treatment is a gluten-free diet that places a social and economic burden on patients and fails, in some, to lead to symptomatic or mucosal healing. Thus, an alternative treatment has long been sought after. Clinical studies on celiac disease have shown an association between the presence of certain microbes and disease outcomes. However, the mechanisms that underlie the effects of microbes in celiac disease remain unclear. Recent studies have employed disease models that have provided insights into disease mechanisms possibly mediated by bacteria in celiac disease. Here, we have reviewed the bacteria and related mechanisms identified so far that might protect from or incite the development of celiac disease. Evidence indicates bacteria play a role in celiac disease and it is worth continuing to explore this, particularly since few studies, to the best of our knowledge, have focused on establishing a mechanistic link between bacteria and celiac disease. Uncovering host–microbe interactions and their influence on host responses to gluten may enable the discovery of pathogenic targets and development of new therapeutic or preventive approaches.

## 1. Introduction

In individuals who carry the human leukocyte antigen (HLA)-DQ2 and/or DQ8 haplotypes, ingestion of gluten can trigger an autoimmune disease, known as celiac disease, characterized by the production of autoantibodies and damage to the small intestinal mucosa [1]. Approximately 40% of the North American and European populations carry either one of these haplotypes [2]; however only 2–3% develop celiac disease, indicating that these HLA variants are necessary but not sufficient for the development of celiac disease, and that other genetic and/or environmental factors are involved.

Several studies have reported differentially abundant intestinal bacteria in at-risk individuals, who carry the HLA-DQ2 and/or DQ8 haplotypes, and celiac disease patients compared with healthy or non-celiac disease controls, although some findings remain inconsistent [3,4]. Unvarying across studies are reports of lower relative abundance of proven beneficial intestinal bacteria, such as *Bifidobacterium* and *Lactobacillus,* and a higher relative abundance of members of the Proteobacteria phylum in duodenal biopsies of children and adults with active or treated celiac disease (on a gluten-free diet) compared with healthy or non-celiac disease groups [4,5,6]. However, it is still unknown whether these bacterial changes are a contributing factor to celiac disease or a consequence of the chronic inflammatory state resulting from an autoimmune response. In recent years, with the improvement of animal and in vitro models capable of replicating the mucosal environment, new studies have provided mechanistic insights into the function of the bacterial microbiota in celiac disease onset and progression [7].

Although excluded from this review, it is relevant to mention that besides bacteria, other microorganisms have been linked to the onset of celiac disease, such as viruses; for instance, reovirus [8,9], and yeast, namely *Saccharomyces cerevisiae* [10,11]. For example, seroreactivity against the latter has been detected in celiac disease patients with mucosal damage [10]. Another study has reported the presence of anti-*Saccharomyces cerevisiae* antibodies (ASCA) in asymptomatic celiac disease patients, indicating detection of these antibodies early on might be predictive of an increased intestinal permeability to dietary antigens and celiac disease onset [11]. ASCA positivity in celiac disease patients might be the result of a non-specific immune response; however, the role of these antibodies in celiac disease remains unknown.

Here, we have reviewed the bacterial-mediated mechanisms described so far in the literature that might protect from or incite the development of celiac disease. We searched the PubMed, Scopus, and Web of Science databases for English-language articles, with no time frame, by using the following medical subject heading terms and keywords: bacteria, intestinal barrier, immunity, celiac disease, and gluten-induced enteropathy. We included studies that used in vitro and in vivo models of disease and indicated a host–bacteria molecular interaction in celiac disease or showed bacterial effects on celiac disease-related mechanisms. We excluded studies with only associations and without the use of disease models. Reference lists of relevant articles were also scanned for additional studies.

## 2. Pathophysiology of Celiac Disease

Celiac disease is an autoimmune disease triggered by ingestion of gluten in individuals who carry the HLA-DQ2 and/or DQ8 haplotypes, leading to production of autoantibodies and mucosal damage in the duodenum, the proximal small intestine [1]. The symptoms can vary widely and range from intestinal to extraintestinal manifestations; for instance, malabsorption and chronic diarrhea to dermatitis herpetiformis and anemia [2]. Some individuals can remain asymptomatic for years, while others present circulating autoantibodies but have normal small intestinal morphology [12]. Celiac disease is also often associated with other autoimmune conditions, such as type one diabetes mellitus and autoimmune thyroid diseases, with the presence of one of these disorders increasing the risk of developing celiac disease [2].

The main known trigger, gluten, is found in wheat, rye, and barley, and is a complex mixture of grain storage proteins. In particular, wheat gluten is composed of gliadins and glutenins that are highly rich in proline and glutamine amino acids [13]. Because of their high proline content, gliadins and glutenins are resistant to full proteolytic degradation by human gastric, pancreatic, and intestinal brush-border enzymes, resulting in incomplete gluten digestion in the gastrointestinal tract [14].

The ensuing gliadin peptides can set off a cascade of events that ultimately lead to autoimmunity in celiac disease patients. One way of doing this is by binding to the CXC motif chemokine receptor (CXCR3) on enterocytes, the intestinal epithelial cells, causing the activation of MyD88 adaptor protein and release of zonulin, a protein that modulates epithelial barrier function [15]. Zonulin activates the protease-activated receptor-2 (PAR-2), found on the apical and basolateral side of enterocytes, through cleavage of part of its extracellular domain, and is then able to trans-activate the epithelial growth factor receptor (EGFR) [16]. This leads to phosphorylation and disassembly of intestinal tight junctions, a complex of proteins that helps to hold enterocytes together, and consequently increases intestinal permeability [15,16].

Gliadin peptides can then translocate, either via the paracellular or transcellular route [17], to the intestinal lamina propria, the layer of loose connective tissue located under the epithelial layer, and are deamidated by tissue transglutaminase 2 (TG2), an enzyme that converts glutamine amino acids to glutamate (or glutamic acid) residues [18]. Deamination by TG2 improves the binding affinity of gliadin peptides to the HLA-DQ2 and/or DQ8 heterodimers in antigen-presenting cells (APCs), such as dendritic cells [19]. The HLA-gliadin peptides complex is then presented to CD4+ T helper cells that recognize it via their cell surface T cell receptors (TCRs) [20,21]. Once activated, these cells can differentiate into Th1/Th17 subsets and release pro-inflammatory cytokines, such as interferon-gamma (IFN-γ), tumor necrosis factor-alpha (TNF-α) and interleukin-17 (IL-17), causing an adaptive immune response that leads to mucosal damage characterized by villous atrophy, crypt hyperplasia and infiltration of intraepithelial lymphocytes (IELs) [22]. T-cells response can also activate and promote the differentiation of B cells into plasma cells that secrete antibodies against deamidated gliadin peptides and the self-antigen TG2 [23], both hallmarks of celiac disease that are used to diagnose it.

**Figure 1:**
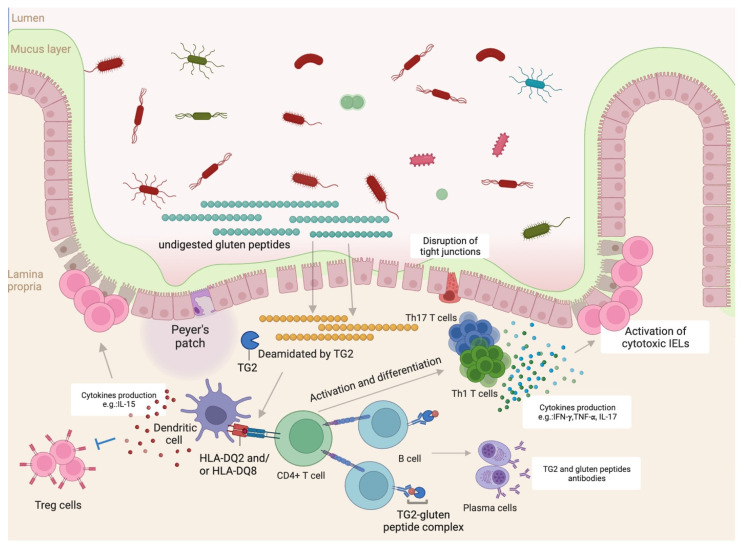
Celiac disease pathophysiology in the proximal small intestine. Schematic representation of disease mechanisms involved in celiac disease development: undigested gluten peptides translocate the epithelial barrier and are deamidated by transglutaminase 2 (TG2) in the lamina propria [18]; these deamidated peptides have increased affinity for human leukocyte antigen (HLA)-DQ2 and/or DQ8 in antigen-presenting cells, such as dendritic cells, that present them to CD4+ T cells [19]; once activated, T cells start producing cytokines, such as interferon-gamma (IFN-γ), tumor necrosis factor-alpha (TNF-α) and interleukin-17 (IL-17) [22]. Antigen-presenting cells also release interleukin-15 (IL-15) that can inhibit regulatory T cells (Treg cells) and increase populations of cytotoxic intraepithelial lymphocytes culminating in tissue destruction [24,25]. Figure created with BioRender.com.

Gliadin peptides, in celiac disease patients, can also cause the secretion of interleukin-15 (IL-15) from enterocytes and dendritic cells. IL-15 can inhibit regulatory CD4+ T (Treg) cells, increase the number of IELs with a cytotoxic phenotype in celiac disease patients, and is also required for the development of villous atrophy [24,25] (Figure 1).

A single trace of gluten can cause the abovementioned immune response and intestinal mucosal damage, causing the gluten-free diet, the current and sole treatment for celiac disease, to be demanding for celiac disease patients. This diet also places an economic and social burden on patients and might fail to improve symptoms in about 20–50% of patients [2]. Recent research has focused on finding alternative or additional treatment options for celiac disease patients [26].

## 3. Intestinal Microbiota Alterations and Host Responses to Gluten in Celiac Disease

The intestinal microbiota influences nutrient absorption, barrier function, and mucosal immune maturation [27]. In particular, members of the intestinal microbiota have different effects on the host’s adaptive immune system and thus can cause variability in immune responses and susceptibility to autoimmune disorders.

One study showed that in the absence of microbes, long-term application (since birth) of gliadin to germ-free AVN-strain Wistar rats led to morphological changes in the small intestine, including crypt hyperplasia, higher cell proliferation in the epithelium crypts, and an increased number of IELs compared with controls fed human serum albumin [28].

A later study reported a similar result in germ-free non-obese diabetic (NOD) mice expressing the human HLA-DQ8 gene that were sensitized with a pepsin–trypsin digest of gliadin (PT-gliadin) and the mucosal adjuvant cholera toxin [29]. Oral gavage of gluten led to increased epithelial cell death and a higher number of IELs, with increased expression of NKG2D and granzyme B in CD3+ *β*TCR+ IELs. NKG2D and granzyme B mediate epithelial cell death and are increased in IELs from active celiac disease patients [30,31]. Germ-free NOD/DQ8 mice also developed gliadin-specific antibodies and T-cell response that was absent in NOD/DQ8 mice colonized with a clean specific-pathogen-free (SPF) bred in gnotobiotic conditions with a microbiota free from opportunistic, pathogenic bacteria and Proteobacteria and dominated by members of Bacteroidetes and Firmicutes phyla. On the contrary, NOD/DQ8 mice with a conventional SPF, which harbors a microbiota with opportunistic pathogens from the Proteobacteria phylum, as *Helicobacter* and *Escherichia* species, developed increased IELs and decreased villus to crypt ratios upon gluten challenge in comparison to clean SPF mice. Additionally, when the NOD/DQ8 mice with conventional SPF were treated perinatally with the antibiotic vancomycin, resulting in lower fecal microbiota diversity and an increase in the relative abundance of Proteobacteria, the numbers of IELs increased. Similarly, when an enteroadherent strain of *Escherichia coli* from a celiac disease patient was administered to clean SPF mice, they exhibited higher IELs counts and gliadin-specific CD4+ T-cell proliferation after gluten challenge [29].

Host immune responses to gluten seem not only to be influenced by the presence or absence of microbes but by the relative abundance of each member of the gut microbiota.

Another study used HCD4/DQ8 mice sensitized with gluten and orally administered indomethacin, a non-steroidal anti-inflammatory drug, to induce intestinal barrier dysfunction and potentiate the intestinal mucosal effects of gluten [32]. Indomethacin induced a higher increase in permeability and an alteration in the structure of the tight junctions in gluten-sensitized mice compared with mice that were non-sensitized, gluten-sensitized only or treated only with indomethacin. These mice also had the most predominant alterations in the intestinal microbiota, with a significant reduction in bifidobacteria and members of the *Clostridium leptum* group, as well as an increase in IgM antibody responses to commensal bacterial antigens. These data indicate that permeability increased by an external factor, in genetically predisposed individuals, might disrupt the commensal community, thus affecting homeostasis and promoting a host pro-inflammatory response to gluten. However, it is still unclear whether these microbial changes are a result of or a contributing factor to the effects seen in this model.

An additional study challenged ex vivo small intestinal biopsies of treated celiac disease patients with trypsin-digested gluten and bacterial isolates from the small intestine of celiac disease patients: four *Prevotella* isolates, one *Lachnoanaerobaculum umeaense* isolate, and one *Actinomyces graevenitzii* isolate [33]. The authors found that this mixture of bacteria, during gluten challenge, either heightened or suppressed the IL-17A, possibly indicating that alterations to the underlying composition of the resident microbiota might influence the immune response to gluten.

Results from these studies using disease models relevant to the study of celiac disease support the role of microbes in the pathogenesis of celiac disease, though the mechanisms and direct causality remain unproven.

## 4. Harmful Effects of Pathobionts and Pathogenic Bacteria in Celiac Disease

Several studies have found that bacteria from the human gastrointestinal tract are capable of degrading gluten peptides resistant to digestion by human enzymes. *Bacteroides* spp., intestinal commensal organisms, have been linked to mucosal immunity regulation but also disease development, including celiac disease. In Caco-2 cell cultures, *B. fragilis* strains carrying metalloprotease virulence genes generated immunogenic peptides from gliadin digestion that disrupted intestinal tight junctions and increased intestinal permeability, making the mucosa more permeable to these peptides [34].

Likewise, in a study using a gnotobiotic approach, *Pseudomonas aeruginosa*, isolated from the duodenum of celiac disease patients, produced highly immunogenic gluten peptides, through the production of LasB, or elastase B, a metalloprotease involved in gluten degradation [35]. The authors found that *P. aeruginosa* cleaved the 33-mer peptide from alpha(α)-gliadin into peptides of 10 to 30 amino acids. The 33-mer peptide, which results from incomplete gluten digestion by mammalian enzymes, is considered the most immunodominant gluten peptide containing six overlapping T-cell epitopes [14]. These smaller peptides produced by *P. aeruginosa* induced an autoimmune response as strong as the 33-mer peptide in gluten-specific T-cells in HLA-DQ2.5+ celiac disease patients, as measured by the production of IFN-γ. Further digestion of these peptides into 4 to 12 amino acids peptides by a bacterial community, from healthy subjects, dominated by *Lactobacillus* spp. decreased their ability to activate an inflammatory T-cell response. These data indicate that the presence of certain microbes in the intestinal microbiota might mitigate the harmful effects of coexisting pathobionts.

The authors also measured the translocation of gluten peptides in mouse jejunum and found that prior incubation of PT-gliadin with *P. aeruginosa* led to an increased translocation of gliadin peptides across the mucosal barrier compared with sole PT-gliadin. This indicates that further digestion by pathobionts of gluten peptides in the small intestine might favor their translocation and the ensuing cascade of events that ultimately leads to autoimmunity in celiac disease.

A subsequent study demonstrated that LasB cleaves the external domain of a host receptor, PAR2, indicating it might act through its activation and ensuing inflammatory signaling [36]. LasB was sufficient to promote a pro-inflammatory response of IELs of the duodenum, with the cleavage of the PAR-2 receptor key to triggering this response.

*P. aeruginosa* LasB was also shown to degrade cathelicidin-related antimicrobial peptide (CRAMP), the mice ortholog of LL-37 in humans [37]. CRAMP is an antimicrobial peptide produced by epithelial cells and infiltrating immune cells. Mice with gluten-induced enteropathy were defective in CRAMP production and had worse clinical features of disease, loss of duodenal tight junctions, increased zonulin production and, consequently, increased intestinal permeability. These improved once CRAMP was exogenously administered, restoring the levels of expression of genes involved in tight junction formation and zonulin production. The authors showed that CRAMP inhibited gliadin-triggered phosphorylation of EGFR and downstream effects.

Similarly, in Caco-2 cells, LL-37 acted through competitive binding to abrogate prolonged activation of EGFR by gliadin [37]. The authors also noted that LasB was increased in sensitized mice on a gluten-containing diet and that CRAMP was negatively associated with *P. aeruginosa*. By supplementing the mice with two virulent strains of *P. aeruginosa*, they observed an increase in LasB in duodenal tissue corresponding to reduced CRAMP. Antibiotic treatment was able to increase CRAMP production in the serum and duodenum and alleviated barrier damage and immune dysregulation in the epithelium. These results indicate that alterations to the composition of the microbiota, with *P. aeruginosa* and other opportunistic pathogens colonizing the small intestine, may constitute an additional factor in celiac disease.

Other Proteobacteria, like *E. coli* and *Shigella* can influence cytokine production after gliadin stimulation when co-cultured with peripheral blood mononuclear cells and dendritic cells. The *Shigella* strain had a higher pathogenic effect than the *E. coli* strain, which potentially links their pathogenic potential to the variation in inflammatory states in celiac disease and could constitute an additional risk factor [38]. These strains were also shown to increase intestinal permeability and gliadin peptide translocation in intestinal loops [39]. Increased virulence and translocation of these bacteria through the epithelial barrier could contribute to celiac disease, although the exact mechanisms remain unclear.

## 5. Beneficial Effects of Commensal Probiotic Strains and Their Metabolites in Celiac Disease

While some taxa degrade gluten and produce highly immunogenic peptides, others are capable of detoxifying gluten peptides. A combination of bacterial strains of *Lactobacillus* and *Bifidobacterium* was shown to hydrolyze the gliadin peptide 33-mer that results from gluten digestion by pepsin and trypsin [40]. The complementary effects of these strains in vitro on Caco-2 cell lines were able to generate low-molecular-weight peptides and thwart the release of cytokine IL-6 and differentiation of cytotoxic T cells.

In a study performed using peripheral blood mononuclear cells, *Bifidobacterium longum* and *B. bifidum* strains isolated from the stool of healthy babies reversed the proinflammatory effects of fecal microbiota obtained from celiac disease patients, increasing the production of IL-10, an anti-inflammatory cytokine that inhibits Th1 cell activation [41].

In a subsequent study using Caco-2 cell lines, these strains decreased the effect of gliadin peptides on initiating NF-κB p65 signaling pathway, TNF-α and IL-1b production by degrading gliadin peptides to a less cytotoxic effect [42]. Similar results were found in a mouse model capable of reproducing a CD4+ T-cell-mediated enteropathy in the presence of gliadin, where colonization by *B. longum* CECT 7347 strain increased levels of IL-10 and reduced CD4+ T cells, counteracting the effects of gliadin [43]. Moreover, the use of this strain in Caco-2 cells in the presence of gliadin increased cell viability and abrogated alterations in proteins otherwise increased by the proinflammatory effects of gliadin [44]. Examples include proteins involved in the cellular signaling of gliadin, like regulator of G-protein signaling 5, proteins of the actin filaments and cycle progression and proteins involved in the crosstalk between intestinal and immune cells, like sorting nexin-20 and T-cell receptor R chain V region CTL-L17 [44].

In HLA-DQ8 transgenic mice sensitized with gliadin, capable of inducing a Th1-like mucosal response, *Lacticaseibacillus casei,* formerly named *Lactobacillus casei,* administration was able to partly recover the mucosal damage caused by gliadin-induced enteropathy, namely a recovery of villus blunting, and reduce levels of TNF-α [45].

Overall, these results show how specific bacterial strains might exert protective benefits by counteracting the host responses driven by gluten peptides or by further digesting these peptides to a less immunogenic state, but the mechanisms remain unclear.

Another way these bacteria might influence celiac disease development is through the production of aryl hydrocarbon receptor (AhR) ligands. AhR is activated in several cell types by many indole-containing ligands resulting from tryptophan metabolism, including those produced by the gut microbiota, and regulates different aspects of the host immune response. A recent study has also found that AhR inhibits activation of the actin regulatory proteins MyoIIA and ezrin when activated by tryptophan ligands produced by gut bacteria [46]. This helps to maintain intact the tight and adherens junctions, which hold together the enterocytes, and therefore the barrier integrity, reducing intestinal permeability.

In the intestinal mucosa of active celiac disease patients, AhR expression is downregulated. A study found that these patients had less AhR ligands in stool, and their intestinal microbiota had a decreased effect on activating this receptor when compared with non-celiac individuals [47]. Using mice expressing the HLA-DQ8 susceptibility gene, the authors were able to modulate the intestinal microbiota by providing an enriched tryptophan diet, leading to AhR ligand production and further receptor activation, and thus improving gluten immunopathology. This was also observed when adding *Limosilactobacillus reuteri,* formerly named *Lactobacillus reuteri,* strains capable of producing AhR ligands. Previously, a study using intestinal organoids co-cultured with lamina propria lymphocytes had found that a metabolite produced by another strain of *L. reuteri* lead lamina propria lymphocytes to produce IL-22 through AhR, accelerating proliferation of intestinal stem cells and recovery of intestinal epithelia upon damage with TNF-α [48]. Both studies also show that these protective effects might be strain-specific and highlight the importance of identifying and characterizing intestinal bacterial strains before their use in therapeutic interventions.

Another beneficial bacterial mechanism has been described as involving a commensal *B. longum* strain, *B. longum* NCC2705, isolated from a healthy infant. In a DQ8 mouse model of gluten sensitivity, it was shown that the beneficial effect exerted by the bacteria was mediated by the production of a serine protease inhibitor, also known as serpin, that resulted in the prevention of gliadin-induced immunopathology [49].

Besides serpins, other microbial products have shown modulating effects in the epithelial barrier and in the immune system. Freire et al. (2019) have demonstrated that celiac disease patient-derived organoids respond differently to gliadin and improve barrier function when treated with bacterial metabolites such as lactate and butyrate, and with *Bacteroides fragilis* polysaccharide A, a capsular exopolysaccharide. These metabolites and the capsular component increased the level of expression of genes coding for mucins, trefoil factor 1 (TFF1) and claudin 18 (CLDN18) that were found to be expressed at a lower rate in celiac disease organoids [50].

Another study has shown a direct link between the alternative splicing in FOXP3 isoforms and the beneficial bacterial metabolite butyrate [51]. FOXP3 is a key transcription factor controlling T-cell development and function with several splicing variants in humans. Deficiency in FOXP3 determines highly aggressive systemic autoimmunity, both in mice and in humans. In this study, the authors have shown that butyrate coupled with IFN-γ dictates the balance between FOXP3 isoforms, FOXP3 ∆2 and full-length FOXP3, in the intestine of non-celiac controls, contrary to what happens in celiac disease patients, in whom lactate increases both isoforms [51]. These results indicate that epigenetic changes led by the microbiota and their respective metabolites could contribute to the immune response in celiac disease, and further studies are needed to explore and fully understand the mechanisms involved.

## 6. Conclusions

Host–microbe interactions have a critical role in maintaining intestinal homeostasis by influencing host immunity. Disruption of these interactions could contribute to the breakdown in intestinal homeostasis and to autoimmune diseases. Cell and animal models of celiac disease have provided proof-of-concept studies indicating a link between host responses to gluten and the composition of the intestinal microbiota and its metabolites. However, it remains unclear whether these changes in bacterial composition precede or follow the host inflammatory and immune response to dietary gluten. Another consideration to be drawn is that of strain specificity, since, for instance, not all strains can produce AhR ligands or degrade gluten peptides. Additionally, few studies have focused on exploring the role of bacterial communities and neglected microbe–microbe interactions that could lead to a disturbance of intestinal homeostasis. Overall, the continuous improvement of animal and cell models can provide clues into the mechanisms of disease possibly influenced by the human microbiota. The discovery of pathogenic targets could provide a ground for the development of novel therapeutics to prevent or treat celiac disease.

## Data Availability

Not applicable.
